# IgG4-Related Autoimmune Prostatitis: Is It an Unusual or Underdiagnosed Manifestation of IgG4-Related Disease?

**DOI:** 10.1155/2013/295472

**Published:** 2013-11-17

**Authors:** María T. Bourlon, Mónica Sánchez-Ávila, Fredy Chablé-Montero, Ricardo Arceo-Olaiz

**Affiliations:** ^1^Medicine Department (Medical Direction), National Institute of Medical Sciences and Nutrition Salvador Zubirán (INCMNSZ), Mexico City, Vasco de Quiroga 15, Col. Sección XVI, 14000 México, DF, Mexico; ^2^Infectious Disease Department, National Institute of Medical Sciences and Nutrition Salvador Zubirán (INCMNSZ), Mexico City, Vasco de Quiroga 15, Col. Sección XVI, 14000 México, DF, Mexico; ^3^Pathology Department, National Institute of Medical Sciences and Nutrition Salvador Zubirán (INCMNSZ), Mexico City, Vasco de Quiroga 15, Col. Sección XVI, 14000 México, DF, Mexico; ^4^Urology Department, National Institute of Medical Sciences and Nutrition Salvador Zubirán (INCMNSZ), Mexico City, Vasco de Quiroga 15, Col. Sección XVI, 14000 México, DF, Mexico

## Abstract

IgG4-related disease (IgG4-RD) encompasses a wide range of extrapancreatic manifestations. Albeit some are relatively well known, others such as autoimmune prostatitis remain poorly described. We present a 61-year-old Latin-American male with autoimmune pancreatitis (AIP) who presented with lower urinary tract symptoms (LUTS), normal prostate specific antigen (PSA) test, and prostate enlargement attributed to benign prostatic hyperplasia (BPH). He underwent a transurethral resection of the prostate (TURP) after which symptoms were resolved. On histopathology, prostatic stroma had a dense inflammatory infiltrate rich in plasma cells and lymphocytes; immunohistochemical morphometric assessment showed >10 IgG4-positive plasma cells/high power field (HPF). The diagnosis of IgG4-related prostatitis was postoperatively. We compared the patient characteristics with those of previous reports on Asian patients. Shared findings included prostate enlargement, LUTS (symptoms that can be confused with BPH), and PSA within normal limits or mild elevations. IgG4-related prostatitis is rarely considered as a preprocedural diagnosis, even in patients with evidence of IgG4-RD. Involved prostate zones include mainly central and transitional zones and less frequently the peripheral. Currently, there is insufficient data about the natural history and outcome. Whether steroids, transurethral resection, or both are the treatment of choice needs to be elucidated.

## 1. Introduction

Increasing interest on the study of autoimmune pancreatitis (AIP) has led to the characterization of several extrapancreatic manifestations [1, 2]. Within this context, the concept of IgG4-related disease emerges. Association with cholangitis [3], sialadenitis [4], lymphadenopathy [5], retroperitoneal fibrosis [5], interstitial nephritis [6], pneumonitis, and thyroid disease [7] has been recognized worldwide. IgG4-related autoimmune prostatitis remains one of the less studied entities, and available evidence is limited to case reports mainly on Asian population [8–10]. Mostly diagnosis is on histopathology, since clinical manifestations are mainly LUTS, and differential diagnosis primarily includes BPH and prostate cancer (PC) [8, 10].

## 2. Case Presentation

A 61-year-old Latin-American male with past medical history of LUTS attributed to BPH was treated with alpha-blockers during 5 years with symptoms remission. He presented with significant weight loss (13 kg/one month), nonspecific abdominal pain, and biliary tract obstruction secondary to a fibrotic pancreatic-duodenal mass noted during an exploratory laparotomy. He suffered from several episodes of acute pancreatitis with undetermined etiology during the following year. An abdominal computed tomography (CT) scan showed diffuse pancreatic swelling, and endoscopic ultrasound (US) biopsy resulted in chronic inflammation with common bile duct fibrosis. Because of persistent abdominal pain, a second exploratory laparotomy was performed in which a peripancreatic tumor was found and a biliodigestive derivation was carried out. Histopathological features revealed chronic pancreatitis with periductal lymphoplasmacytic infiltrate without granulocyte infiltration consistent with the diagnosis of autoimmune pancreatitis. Laboratory exams showed hypergammaglobulinemia and positive antinuclear antibodies (ANAs), and serum IgG4 was 32 mg/dL (normal reference range: 1–115 mg/dL).

During followup, evidence of autoimmunity in other systems aroused. He received the diagnosis of vitiligo and persistent elevation of serum IgG. He had xerostomia and xerophthalmia with a negative Schirmer test and gland scintigraphy with mild dysfunction of submaxillary glands. Episodes of painful axillary and cervical lymphadenopathy associated with significant weight loss and arthralgias appeared. LUTS worsened throughout the following months. Transrectal ultrasound noted a prostate enlargement. Levels of PSA always remained within normal limits 0.15–1.62 ng/mL (normal reference range: 0.0–4.0 ng/mL).

**Figure 1:**
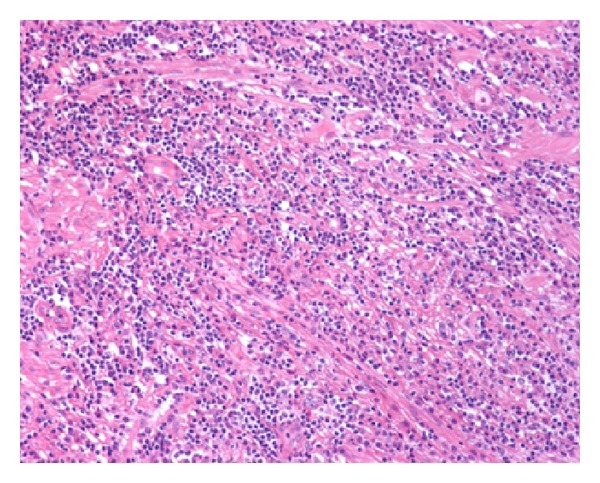
Low power view of IgG4-related prostatitis. The prostatic stroma shows a dense inflammatory infiltrate and fibrosis (H&E, 100x).

**Figure 2:**
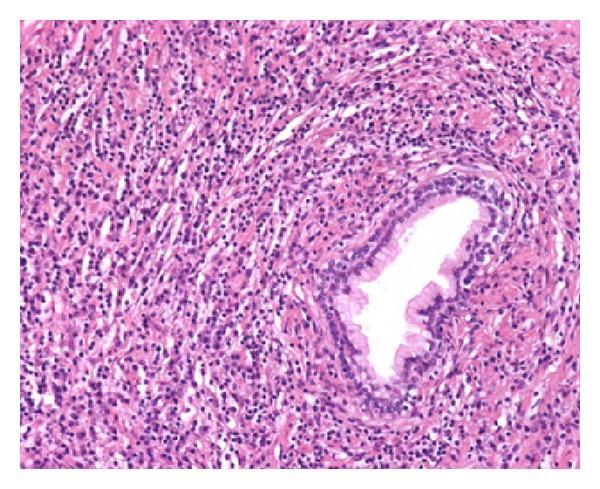
IgG4-related prostatitis. A prostatic gland is surrounded by a dense inflammatory infiltrate with numerous plasma cells. Prostatic vessels showed obliterative phlebitis (H&E, 200x).

**Figure 3:**
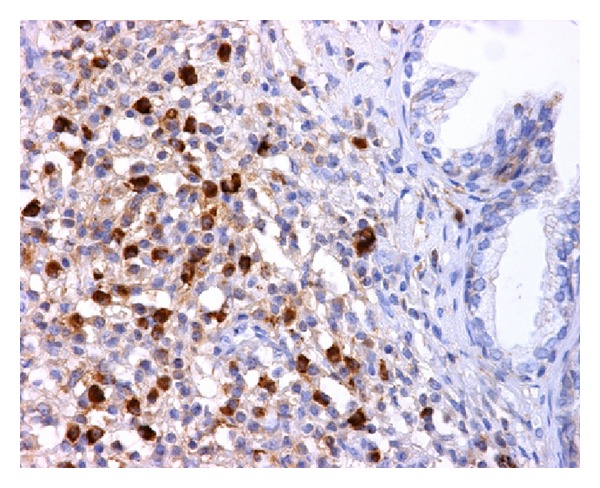
IgG4-related prostatitis. Numerous IgG4-positive plasma cells are shown (>10 cells/HPF) (Immunomarker against IgG4, 400x).

He underwent a transurethral resection of the prostate (TURP) due to the persistence of moderate LUTS despite adequate medical treatment under the suspicion of BPH. Numerous fragments of prostatic tissue underwent analysis. The specimen weighed 25 grams. On light microscopy, prostatic stroma had a dense inflammatory infiltrate rich in plasma cells and lymphocytes (Figure 1). In some areas, the inflammatory elements surrounded atrophic as well as normal prostatic glands (Figure 2). Prostatic vessels showed obliterative phlebitis. On immunohistochemical morphometric analysis >10 IgG4-positive plasma cells/HPF were encountered (Figure 3). Postoperatively, the diagnosis of IgG4-related prostatitis was established. After the transurethral prostate resection, the patient has remained asymptomatic.

## 3. Discussion

Data on IgG4-RD involving the lower urinary tract is limited. It is restricted to case reports and a small case series to which we compared this case [8–10] (Table 1). Reported patients have all been Asian, and the present constitutes to our knowledge the first Latin-American documentation. Commonly, men in the sixth and seventh decade of life are affected. This group of age overlaps with the target group affected by BPH and PC, making the differential diagnosis even more challenging. The majority of cases share a history of either synchronous or metachronous IgG4-RD manifestations, mainly with involvement of the pancreas [8, 10]. However, IgG4-RD prostatitis is seldom if ever the pre-procedural diagnosis.

Misdiagnosis of IgG4-RD with malignancy is frequently encountered [12]. Given that swelling occurs in the infiltrated organs, AIP has been falsely diagnosed as pancreatic cancer, Mickulicz's disease mistaken with lymphoproliferative disorders and myofibroblastic tumors misguided for neoplasms [12].

Nonetheless, association of IgG4-RD and malignancy has been described [13]. From the cases reported to date of IgG4-related prostatitis, only one had PC simultaneously [10]. Some cases have debuted with PSA elevation. The diagnosis of prostate cancer is assumed with no further confirmation, leading to an increase the misdiagnosis.

The natural history of the disease leads to fibrotic tissue replacement in different glandular structures [5, 12]. Most patients with IgG4-RD prostatitis described in the literature had acinar atrophy [8–10]. Therefore, scarce normal prostatic tissue could explain low PSA levels in these subjects.

IgG4-RD, whether AIP or with organ involvement elsewhere, has a clear tendency to be effectively treated with corticosteroids [1, 2, 5]. Prompt recognition of this disease may lead to consider the decision of glucocorticoid administration. Nearly all patients develop symptoms easily confounded with BPH, such as, frequency, nocturia, weak urinary stream, hesitancy, intermittence, incomplete emptying, and urgency. Some have undergone, just as our patient, TURP with favorable outcomes [9]. The International Prostate Symptom Score (IPSS) is a good tool to assess LUTS severity and clinical outcomes after treatment in patients with BPH and could help to objectify treatment followup in this scenario [10]. Descriptions on metabolic activity on fluorodeoxyglucose positron emission tomography (FDG-PET) as a good response parameter have been described in patients on steroids [8].

Limited evidence is available to judge which prostatic zones are involved. Given that documentation of our case was on specimens from TURP, only the central and transitional zones were subject to analysis. Both were found to have infiltration by lymphocytes and plasmatic cells. Previous reports in which patients have undergone radical prostatectomy reported diffuse inflammatory changes in the periphery as well [8, 9]. Nevertheless, involvement of central and transitional zones is what leads to patient's LUTS. Prostate, as other affected organs, has lymphocytes and plasma cells infiltrations showing strong immunoreactivity to IgG4 and severe atrophy of exocrine glands with dense fibrosis [8–10]. This fact supports the hypothesis that the pathogenic mechanism herein is present among the wide spectrum of the disease.

A noticeable finding when comparing data is that our case stands out for being the only report with normal serum IgG4. Given that this is an isolated case report, no conclusions can be stated. A possible explanation is that IgG4 as a serum marker may fluctuate according to disease activity. Systematic measurement of IgG4 serum levels instead of an isolated determination may have yielded distinct results. As other serum markers, it is as a helpful tool but not a synonym of disease diagnosis. Finally, we did the diagnosis on histopathological findings. Systematic measurement of IgG4 serum levels instead of an isolated determination may have yielded distinct results. As other serum markers, it is a helpful tool but not a synonym of disease diagnosis.

After analyzing published data, the inevitable emerging question is whether autoimmune prostatitis is really an unusual manifestation or just an underdiagnosed entity. Even when patients have a clear history of IgG4-RD, clinicians rarely consider this diagnostic possibility when aged men present with LUTS. It is important to highlight that this is a recently described form of the disease. Its natural history is under description. The fact that most initial reports are on Asian patients might reflect a tendency to affect certain ethnicities and thus its high prevalence in this part of the world. More evidence will be published on the disease; case reports on other populations might appear and show this disease to be a more frequent manifestation than it is now believed.

Certainly, clinicians will face autoimmune prostatitis long before the immunopathogenic basis of disease is understood. Recently a patient with an abnormal digital exam and steroid-responsive urinary symptoms due to IgG4-related prostatitis was reported. While sufficient evidence is gathered to define the best treatment modality, physicians will take the challenge to start immunosuppressive medications or indicate procedures such as TURP as an attempt to control symptoms.

## Figures and Tables

**Table 1:** Clinical and pathologic comparison among reported cases of IgG4-related prostatitis.

#	Age	Ethnicity	Evidence of IgG4-RD	Serum IgG4	Symptoms	Clinical Dx	PSA (ng/mL)	Procedure	Rx with steroids	Response to Rx	Time at Dx
Yoshimura et al., 2006 [9]											
1	65	Asian	AIP, sclerosing cholangitis, sialadenitis	↑	Obstructive urinary symptoms, urinary frequency, enlarged prostate	BPH	WNL	TURP	−	I	PO

Nishimori et al., 2007 [8]											
2	64	Asian	AIP	↑	Asymptomatic on admission	BPH	<0.01	NB	+	I	Post-NB
3	67	Asian	—	↑	Prostate tenderness	BPH	1.62	TURP	−	I	PO

Uehara et al., 2008 [10]											
4	65	Asian	AIP	↑	NA	PC	5.5	Radical prostatectomy	−	NA	PO*
5	73	Asian	AIP	↑	Dysuria, prostate enlargement, IPSS: 20	SPC	7.2	NB	−	NA	Post-NB
6	71	Asian	AIP	↑	Dysuria, IPSS: 21	ML	1.57	NB	+	I	Post-NB
7	55	Asian	AIP	↑	Dysuria, prostate enlargement, IPSS: 12	CP	0.38	NB	+	I	Post-NB
8	73	Asian	AIP	↑	Dysuria, prostate enlargement	SPC	5.84	NB	−	NA	Post-NB
9	66	Asian	AIP	↑	Dysuria, prostate enlargement, IPSS: 18	SPC	0.1	NB	+	I	Post-NB

Hart et al., 2013 [11]											
10	55	American	AIP	↑	Enlarged prostate, nocturia, hesitancy	SPC	0.67	NB	+	I	Post-NB

Bourlon et al., 2011 [4]											
11	61	Latin-American	AIP, sialadenitis, LAD	WNL	Obstructive urinary symptoms, prostate enlargement, IPSS: 15	BPH	0.27–1.19	TURP	−	I	PO

AIP: autoimmune pancreatitis; BPH: Benign prostatic hyperplasia; Dx: diagnosis; FDG-PET: positron emission tomography with [^18^F] fluorodeoxyglucose; I: improved; LAD: lymphadenopathy; ML, NA: nonavailable; NB: needle biopsy; PC: prostate cancer; PO: status postoperative; Post-NB: postneedle biopsy; PSA: prostate specific antigen; Rx: treatment; SPC: suspicious for prostate cancer; TURP: transurethral resection of the prostate; WNL: within normal limits. *Concomitant diagnosis of prostate cancer.
